# Developmental Exposure to Bisphenol A Modulates Innate but Not Adaptive Immune Responses to Influenza A Virus Infection

**DOI:** 10.1371/journal.pone.0038448

**Published:** 2012-06-04

**Authors:** Anirban Roy, Stephen M. Bauer, B. Paige Lawrence

**Affiliations:** Department of Environmental Medicine, University of Rochester School of Medicine and Dentistry, Rochester, New York, United States of America; University of California Los Angeles, United States of America

## Abstract

Bisphenol A (BPA) is used in numerous products, such as plastic bottles and food containers, from which it frequently leaches out and is consumed by humans. There is a growing public concern that BPA exposure may pose a significant threat to human health. Moreover, due to the widespread and constant nature of BPA exposure, not only adults but fetuses and neonates are also exposed to BPA. There is mounting evidence that developmental exposures to chemicals from our environment, including BPA, contribute to diseases late in life; yet, studies of how early life exposures specifically alter the immune system are limited. Herein we report an examination of how maternal exposure to a low, environmentally relevant dose of BPA affects the immune response to infection with influenza A virus. We exposed female mice during pregnancy and through lactation to the oral reference dose for BPA listed by the US Environmental Protection Agency, and comprehensively examined immune parameters directly linked to disease outcomes in adult offspring following infection with influenza A virus. We found that developmental exposure to BPA did not compromise disease-specific adaptive immunity against virus infection, or reduce the host’s ability to clear the virus from the infected lung. However, maternal exposure to BPA transiently reduced the extent of infection-associated pulmonary inflammation and anti-viral gene expression in lung tissue. From these observations, we conclude that maternal exposure to BPA slightly modulates innate immunity in adult offspring, but does not impair the anti-viral adaptive immune response, which is critical for virus clearance and survival following influenza virus infection.

## Introduction

Bisphenol A (BPA) is a component of polycarbonate plastics and epoxy resins used in a variety of products including food and beverage containers, electronic appliances, dental sealants, paper currency, and receipts [1]. The widespread use of BPA results in an extremely high prevalence of human exposure; for example, about 93% of the United States population has detectable levels of BPA in their urine or blood [2]. This has generated substantial concern because numerous studies show that BPA acts as “endocrine disruptor,” binding to estrogen and thyroid receptors, and possibly acting via other pathways, to cause variety of deleterious health effects [3]–[6]. Work in animal models has fueled concern about the potential harmful effects of BPA to human health, such as abnormalities in reproductive and brain development, gene expression patterns, as well as body weight and altered social behaviors [7]–[9]. Moreover, a commonality among many of these reports is that BPA exposure during pregnancy or shortly after birth is linked to exacerbated disease later in life. Exposure to endocrine disruptors *in utero* or shortly after birth may be even more detrimental than exposure during adulthood because key developmental processes may be permanently altered, leading to negative consequences that last for the lifetime of the offspring. With regard to BPA, this is clearly a possibility as fetuses and neonates are exposed to BPA from maternal sources, both *in utero* and via breast milk, as well as from BPA that leaches from food containers and other products [1], [10], [11]. Indeed, there is compelling evidence in animal models that maternal exposure to even minute amounts of BPA affects numerous physiological systems in the offspring.

Due to its intricate relationship with the endocrine system, the immune system is a particularly vulnerable target for potentially deleterious effects of maternal exposure to endocrine disrupting agents. Yet, few studies have explicitly examined the relationship between maternal exposure to BPA and altered immune function in the offspring. A handful of epidemiological studies have examined this question, and the findings are mixed, with some finding a possible correlation between urinary BPA levels and metrics of immune function or disease, but other studies not finding a relationship [12]–[15]. Immunomodulatory effects of prenatal BPA exposure, such as a type-2 skewed cytokine response in the offspring have also been reported in several animal studies [16]–[19]. There is significant controversy about the implications of these findings to human health because some effects of BPA were only observed when high doses and/or routes of exposure that are considered less relevant to human exposure were used [20], [21]. Therefore, the consequence of low, environmentally relevant developmental exposures to BPA on the function of the immune system remains uncertain.

Early life exposure to BPA could alter the development and maturation of immune cells, such that key host defense and immunoregulatory mechanisms are impaired later in life. Other possible consequences of early life exposure to BPA include more subtle changes, such as altered functional capacity of leukocytes upon antigen challenge, changes in leukocyte trafficking, and/or cell-to-cell communication pathways that are critical for a properly controlled immune response. In any of these situations, BPA could broadly affect all leukocytes, or selectively modulate the function of a particular lineage. Given that the immune system encompasses an integrated and complex network aimed at finding and eliminating invading pathogens and cancer cells, even subtle impairment could decrease resistance to infection, reduce the efficacy of immunological memory, or impair tumor surveillance mechanisms. Consequently, if developmental exposure to BPA disturbs any of these processes, it would create a situation in which immune responses are imbalanced and responses to challenge are skewed in a way that is less favorable to the host. The potential impact of these types of changes on human health is tremendous.

In the present study, we determined whether maternal exposure to BPA influences the long-term function of the offspring’s immune system using a mouse model of lower respiratory tract infection with human influenza A virus. This model system is useful to assess the developmental immunotoxicity of BPA because we can examine host resistance and quantitatively measure multiple disease-specific endpoints [22], [23]. When influenza viruses enter the respiratory tract, they prompt a series of events that usually lead to successful viral clearance 8–10 days later. The response to infection initially involves innate mediators such as macrophages, neutrophils, cytokines and chemokines, which collectively strive to control viral infection until the adaptive arm of the immune response is fully engaged [24]. The adaptive immune response to influenza virus includes CD4^+^ and CD8^+^ T cells, as well as antibody producing B cells [24]. Of these, the generation of virus-specific cytotoxic CD8^+^ T cells in lymphoid tissues, which emigrate to the lung and kill infected cells, are the principal means for host resistance during a primary viral infection [25], [26]. In fact, CD8^+^ T cells are especially critical for newly emerging strains of influenza virus, where cross-protective antibody mediated responses to viral surface proteins are often absent or less robust. CD4^+^ T cells do not generally kill influenza virus infected host cells directly. Instead, these cells instruct antibody isotype switching in B cells and regulate immunological memory [27], [28]. Virus-specific antibodies provide critical defenses from repeated infection with homotypic virus strains [29]. Thus, insufficient adaptive immunity can result in alterations in the response to primary and repeated infection. We report herein an investigation of the consequences of maternal exposure to the current oral reference dose of BPA on these aspects of the host response to infection. We chose an oral route of exposure to BPA because this represents the major manner through which human exposure occurs [1], and we administered a daily dose that yields systemic BPA levels in the pregnant mice that are similar to levels reported in the general population [2], [12], [30].

## Materials and Methods

### Animals

The specifications for the animal housing environment were based on the guidelines outlined by an NIEHS expert panel for BPA toxicity studies [31]. Adult C57BL/6 mice were purchased from The Jackson Laboratory (Bar Harbor, ME) and housed in pre-washed (BPA-free) polysulfone cages under pathogen free conditions. Mice received phytoestrogen-free irradiated rodent chow (AIN-76A, Test Diet, Richmond, IN) and water that was purified by reverse osmosis and provided in glass bottles. The Environmental Protection Agency reference dose for oral exposure to BPA, which is 50 µg/kg of body weight/day [32], was used in this investigation. Adult mice were paired for breeding, and females were checked daily for vaginal plugs as an indication of pregnancy. The day when a plug was found was defined as the day 0 of gestation. Singly housed, plugged females began receiving 50 µg BPA/kg body weight/day, or vehicle alone (peanut oil) orally starting on gestational day (GD) 6 and continuing daily through postnatal day 21. Each pregnant or lactating female mouse received BPA dissolved in 20 µl of peanut oil absorbed to a single additive-free puffed wheat cereal, or administered by oral gavage in a volume of 200 µl. Each mouse consumes the BPA-spiked food treats in approximately 1 minute under observation, ensuring that an accurate dose is administered to each mouse, on each day, in a stress free manner. On postnatal day 21, all pups were weaned and housed in same sex groups until ready for experimental procedures (conducted when they reached 6–8 weeks of age). All procedures involving laboratory animals were performed in accordance with the protocols approved by the Institutional Animal Care and Use Committee.

### Measurement of BPA

Cardiac blood was collected from a subset of pregnant female mice 4 hours following oral exposure to BPA or peanut oil vehicle. The levels of BPA in blood plasma samples were measured using a high performance liquid chromatography coupled with electrospray triple-quadrupole mass spectrometer [33]. The measurement was done in the Laboratory of Organic Analytical Chemistry, New York State Department of Health at Wadsworth Center, Albany, NY.

### Influenza Virus Infection

Sex matched adult (6–8 weeks old) offspring were intranasally (i.n.) infected with influenza A virus in a final volume of 25 µl of endotoxin-tested PBS under general anesthesia (Avertin, 2,2,2-tribromoethanol; Aldrich, Milwaukee, WI). Both male and female offspring were used, but analyzed separately. For the primary infections in this study, either 120 hemagglutinating units (HAU) of influenza A/HKx31 (H3N2) (hereafter denoted as HKx31) or 50 foci forming units (FFU) of influenza A/CA/04/09 (H1N1) virus was used. For secondary infections, 0.005 HAU of influenza/A/PR/8/34 (H1N1) virus (hereafter denoted as PR8) was used. Infected mice were monitored daily for body weight changes and mortality.

### Lung Histology

Groups of age- and sex-matched mice were sacrificed by anesthetic overdose at the indicated points in time relative to infection. The trachea was surgically exposed, and lungs were fixed with 10% neutral buffered formalin (NBF) *in-situ* at constant pressure under gravity. Fixed lungs were excised and embedded in paraffin. 5 µm thick tissue sections were mounted on glass slides and stained with hematoxylin and eosin (H&E). Histologic scores were based on inflammatory cell accumulation and tissue damages observed under light microscope, as described previously [34]. Briefly, numerical scores were assigned to tissue sections as follows: normal tissue without any cell accumulation  =  0, nominal cell infiltrates restricted to perivascular area  =  1, sporadic cell infiltration into the tissue parenchyma with consistent presence of wide perivascular and peribronchiolar cuffing  =  2, extensive cell infiltration into the parenchyma  =  3.

### Virus Titer Assay

The pulmonary viral burden was measured by determining the number of FFU present in lung homogenates [35]. Briefly, lungs were harvested from infected mice and mechanically homogenized. Serially diluted lung homogenates were incubated overnight on confluent Madin-Darby canine kidney (MDCK) cells in 96 well plates. The next day, plates were washed and foci of infection were detected using biotin-conjugated influenza A virus nucleoprotein specific antibodies (Millipore, Billerica, MA), followed by streptavidin-conjugated alkaline phosphatase and BCIP-NBT as the color developing substrate (Sigma-Aldrich, St. Louis, MO). Foci counts were adjusted to the dilution of lung homogenate to determine the total infectious virus particles in the entire lung; this is denoted in the figures as FFU/lung.

### Analysis of Gene Expression by Quantitative Real Time (qRT)-PCR

Lungs from infected mice were mechanically homogenized on ice and solubilized in Tri reagent (Molecular Research Center, Cincinnati, OH). RNA was extracted using 1-bromo-3-chloropropane (Molecular Research Center) as a phase separation agent, and RNA sequestered in the aqueous layer was purified using RNeasy kits (QIAGEN, Valencia, CA). cDNA libraries were synthesized from 2 µg of total RNA using iScript reagents (BIO-RAD, Hercules, CA). Levels of expression for different genes were assessed by qRT-PCR, using approximately 50 ng of template cDNA, gene specific primers, and BIORAD iQ-SYBR Green Supermix, using a BIO-RAD iQ5. Expression of each gene in a particular sample was normalized to the level of gene expression for a ribosomal protein L-13 (housekeeping gene) in that sample as previously described [36]. Transcript sequences were obtained from NCBI database and primer properties were evaluated using Primer3 software [37]. The primer sequences for all genes used are listed in Table 1.

**Table 1:** Primer sequences for gene expression analysis.

Gene Name	Forward Primer	Reverse Primer
**TNF-α**	CAGGTTCTCTTCAAGGGACAAG	GCAGAGAGGAGGTTGACTTTC
**IL-6**	CCTTCTTGGGACTGATGCTGGT	GACAGGTCTGTTGGGAGTGGTATC
**IFN-β**	AACTATAAGCAGCTCCAGCTCCA	CTGCATCTTCTCCGTCATCTCCA
**IFN-γ**	AGCAACAACATAAGCGTCATT	CCTCAAACTTGGCAATACTCA
**RANTES**	GTGCTCCAATCTTGCAGTCGT	AGGGAAGCGTATACAGGGTCA
**IP-10**	TACTGTAAGCTATGTGGAGGTGCG	AACTTAGAACTGACGAGCCTGAGC
**MCP-1**	TAGGCTGGAGAGCTACAAGAGGAT	CTCTCTCTTGAGCTTGGTGA
**iNOS**	TGGCTACCACATTGAAGAAGCTG	TCTGGCTCTTGAGCTGGAAGAAA
**L-13**	CTACAGTGAGATACCACACCAAG	TGGACTTGTTTCGCCTCCTC

### Characterization of Immune Cells by Flow Cytometry

Lungs, lung-draining mediastinal lymph nodes (MLN), and spleens were removed from infected mice. Lung tissues were digested with collagenase and DNase as described previously [38]. Cells from digested lungs were passed through 70 µm filter to remove clumps and debris. Single cell suspensions of MLNs and spleens were prepared by teasing the tissues apart between two frosted glass slides. Erythrocytes were lysed in all samples using hypotonic ammonium chloride buffer. Cells were washed, counted, incubated with a mixture of rat IgG and anti-mouse CD16/32 antibodies to prevent non-specific binding, and then stained with fluorescent dye-conjugated antibodies against cell surface markers CD3, CD4, CD8, CD25, CD44 and CD62L, and the intracellular protein FoxP3 (eBioscience, San Diego, CA). Virus-specific CD8^+^ T cells were identified using MHC class-I tetramers presenting nucleoprotein (NP_366–374_) and acid-polymerase (PA_224–233_) peptide fragments of influenza A virus. Stained cells were analyzed using an LSR-II flow cytometer (BD Bioscience, Franklin Lakes, NJ) and results were analyzed using FlowJo software (ver. 8.8.7, Tree Star, Ashland, OR).

### Detection of Virus Specific Antibodies

Influenza A virus specific antibodies in blood plasma collected from infected mice were measured using enzyme-linked immunosorbent assay (ELISA) as described before [22]. Briefly, serially diluted plasma samples were incubated in plates coated with inactivated HKx31 virus (Charles River/Spafas, North River, CT). Captured virus specific antibodies were detected using HRP conjugated isotype specific anti-mouse antibodies (Southern Biotech, Birmingham, AL) and H_2_O_2_-fortified ABTS substrate. The absorbance of the substrate was measured at 405 nm using a spectrophotometer.

### Statistical Analyses

Since the dams, not the offspring, were given BPA, the experimental ‘n’ refers to the number of treated dams, not the number of offspring. All sex-matched adult offspring in each treatment group and at each point in time after infection were from a *different* treated dam. Statistical analyses were performed using Prism 4.0 (GraphPad software, La Jolla, CA). For all data sets depicted in each figure, statistical significance of the differences in mean values between the endpoints measured in vehicle versus BPA exposed groups were calculated using the following tests with 95% confidence interval: Mantel-Haenszel log-rank test for survival curves, two-way ANOVA with Bonferroni post-test for body weight changes following infection, and two-tailed unpaired Student’s t-test for all other immunological parameters. An asterisk (*) is used to denote mean values for which the p-value was less than 0.05. The absence of this marking indicates that no statistically significant difference between mean values was observed for that particular data set. Error bars on all graphs represent standard error of the mean (SEM).

## Results

### Maternal Exposure to BPA did Not Change the Body Weight of Either Pregnant Dams or the Offspring

**Figure 1:**
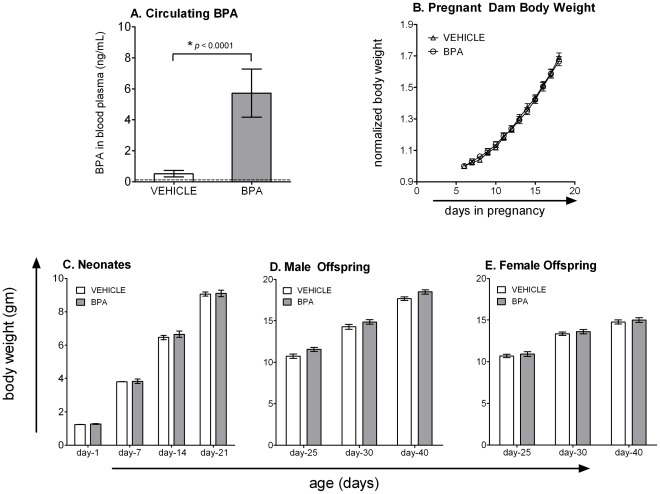
BPA treatment does not change body weights of pregnant dams or the offspring. Pregnant dams were fed BPA spiked treats every day starting on the 6^th^ day of gestation and continuing until 21 days after parturition (when the pups were weaned). Control dams received the same daily treat, but spiked with the peanut oil vehicle. (A) On 16^th^ day of pregnancy a subset of dams were sacrificed 4 hours after eating spiked treats or being gavaged with BPA (or vehicle), and the levels of BPA were measured in blood (limit of detection  =  0.05 ng/mL; dashed line). (B) Dams were weighed daily during pregnancy, and (C-E) the offspring were weighed on postnatal days 1, 7, 14 and 21. Pups were weaned on post-partum day-21 into same sex groups and weighed on regular intervals until the age of 40 days. Data represent mean ± SEM (n≥20/group).

Circulating BPA in pregnant mice was measured to validate exposure from our method of oral delivery of BPA. Measurements of BPA in blood (Fig. 1A) indicate that treated dams had circulating BPA levels within the range reported in the general human population [2], [12], [30]. Moreover, BPA levels in dams given the peanut oil vehicle were significantly lower. Given that BPA is a known endocrine disruptor and has been suggested to be an obesogen [6], [39], [40], we monitored the body weight change of the dams for the duration of pregnancy, starting from the first day of BPA treatment (day 6 after detection of a vaginal plug) until delivery. We also monitored sex ratios and body weight of the offspring from birth to adulthood. We did not observe any differences in the body weight of the dams, or their offspring when comparing the vehicle and BPA treated groups (Fig. 1B & 1C). Likewise, there were no differences in neonatal survival, litter size, or the ratio of male-to-female offspring (data not shown).

### Developmental Exposure to BPA did Not Alter the Primary Outcomes of Influenza A Virus Infection

**Figure 2:**
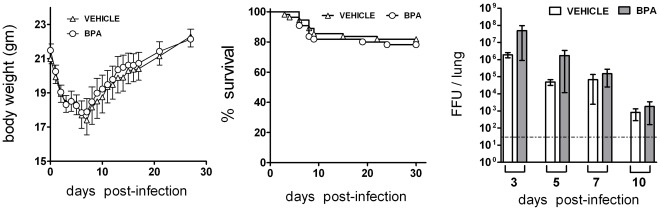
Developmental exposure to BPA does not alter primary disease outcomes from infection with influenza A virus. Developmentally exposed adult offspring were infected with influenza virus (HKx31). All mice were monitored for (A) body weight loss and (B) mortality for a period of at least 30 days post infection (n = 55/group). (C) To determine pulmonary viral burden, sub-groups of infected mice were sacrificed 3, 5, 7 or 10 days after infection and lungs were harvested (n≥5/group/time point). Viral FFU per lung per animal was determined by incubating lung homogenates on MDCK cells, as described in the Material and Methods section. The gray dotted line represents the limit of detection for the assay. Data represent mean ± SEM, and depict findings in female offspring. Parallel assessments were conducted in male adult offspring, and yielded similar results (data not shown).

To examine the immunological competence of developmentally exposed adult offspring, we infected them with influenza virus (strain A/HKx31; H3N2) and examined a set of primary and secondary disease outcomes in female and male adult offspring. Primary outcomes included survival, changes in body weight as a measure of morbidity, and the amount of virus in the lung. Secondary outcomes included pulmonary inflammation, expression of key anti-viral genes, as well as virus-specific CD8^+^ T cell and antibody responses after primary and secondary infection. Following primary infection, we did not observe any differences in mortality, body weight change, or virus clearance from the lung, when we compared mice that were developmentally exposed to BPA to age- and sex-matched offspring of control-treated dams (Fig. 2). Given that influenza A virus strain HKx31 is generally considered to cause a relatively mild infection, we infected a separate group of developmentally exposed mice with a more lethal strain: influenza A virus CA/04/09 (H1N1). While this caused ≥ 50% mortality, we observed no difference in morbidity or mortality in the BPA- versus control-exposed groups (data not shown).

### Maternal BPA Exposure Altered Infection-associated Changes in Lung Inflammation and Gene Expression

**Figure 3:**
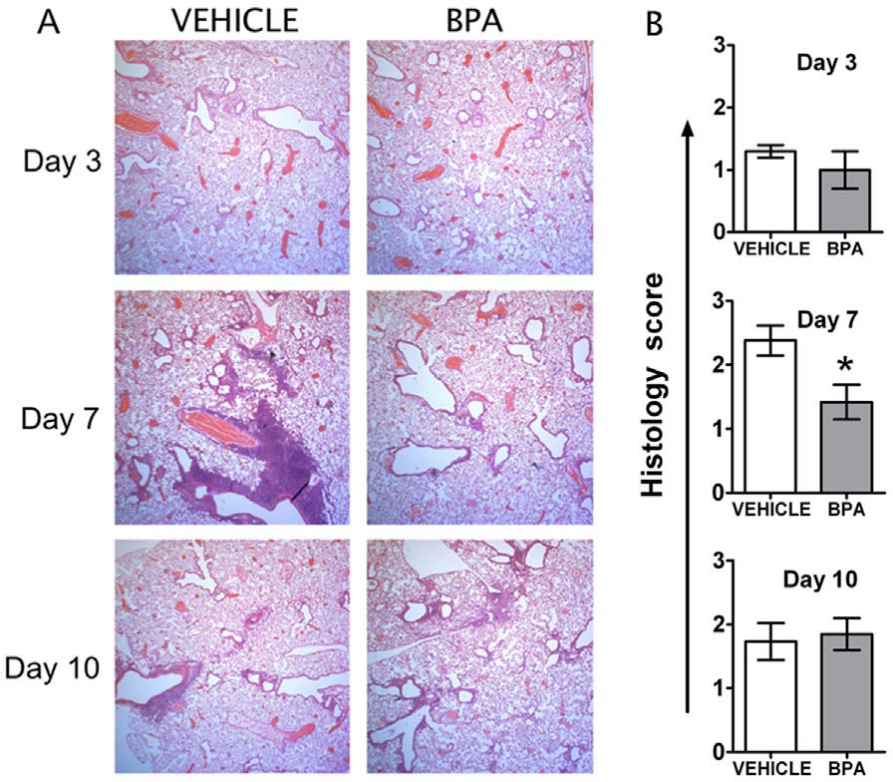
Maternal BPA exposure alters the tissue pathology following influenza A virus infection. Adult offspring of treated dams were infected with influenza virus (HKx31). To examine lung pathology, sub-groups of infected mice were sacrificed 3, 7 or 10 days following infection and lungs were fixed in-situ with 10% neutral buffered saline. Paraffin embedded tissues were cut in 5 µM think sections and stained with H&E. (A) Representative images of tissue sections from infected female mice are depicted. All images are at the same magnification. (B) Tissue sections from all mice in each group were scored based on the degree of inflammatory cell accumulation, as outlined in the Material and Methods section. Data in the graphs represent mean ± SEM (n = 5/group/time point); *p≤0.05.

The equivalent capacity for virus clearance suggested that developmental exposure might not overtly compromise aspects of immune function crucial for surviving infection. This idea is relevant because the global burden associated with influenza virus stems less from mortality and more from the impact of infection-related illness, resulting co-morbidities, and lost productivity. Two aspects of the host immune response that often influence the degree of illness are pulmonary inflammation and mediators of anti-viral immunity [41], [42]. Thus, to further evaluate whether developmental exposure to BPA could have subtle effects on the immune system, we examined pulmonary inflammation and cytokine/chemokine gene expression levels in infected lung tissues. We compared lung inflammation in mice developmentally exposed to vehicle or BPA at three critical times following influenza A virus infection: 1) during the peak of virus replication in the lung (day 3 post-infection [p.i.]), 2) during early virus clearance phase (day 7 p.i.), and 3) during the peak of CD8^+^ T cell mediated response, which ultimately clears virus from the lung and generally dictates the overall outcome of the disease (day 10 p.i.). As shown in Fig. 3, mice that were developmentally exposed to BPA or vehicle control exhibited a similar degree of bronchopulmonary inflammation the early stages of infection. However, there was significantly less severe pulmonary inflammation 7 days after infection. By day 10 p.i., the extent of inflammation returns to an equivalent level in both groups of mice.

**Figure 4:**
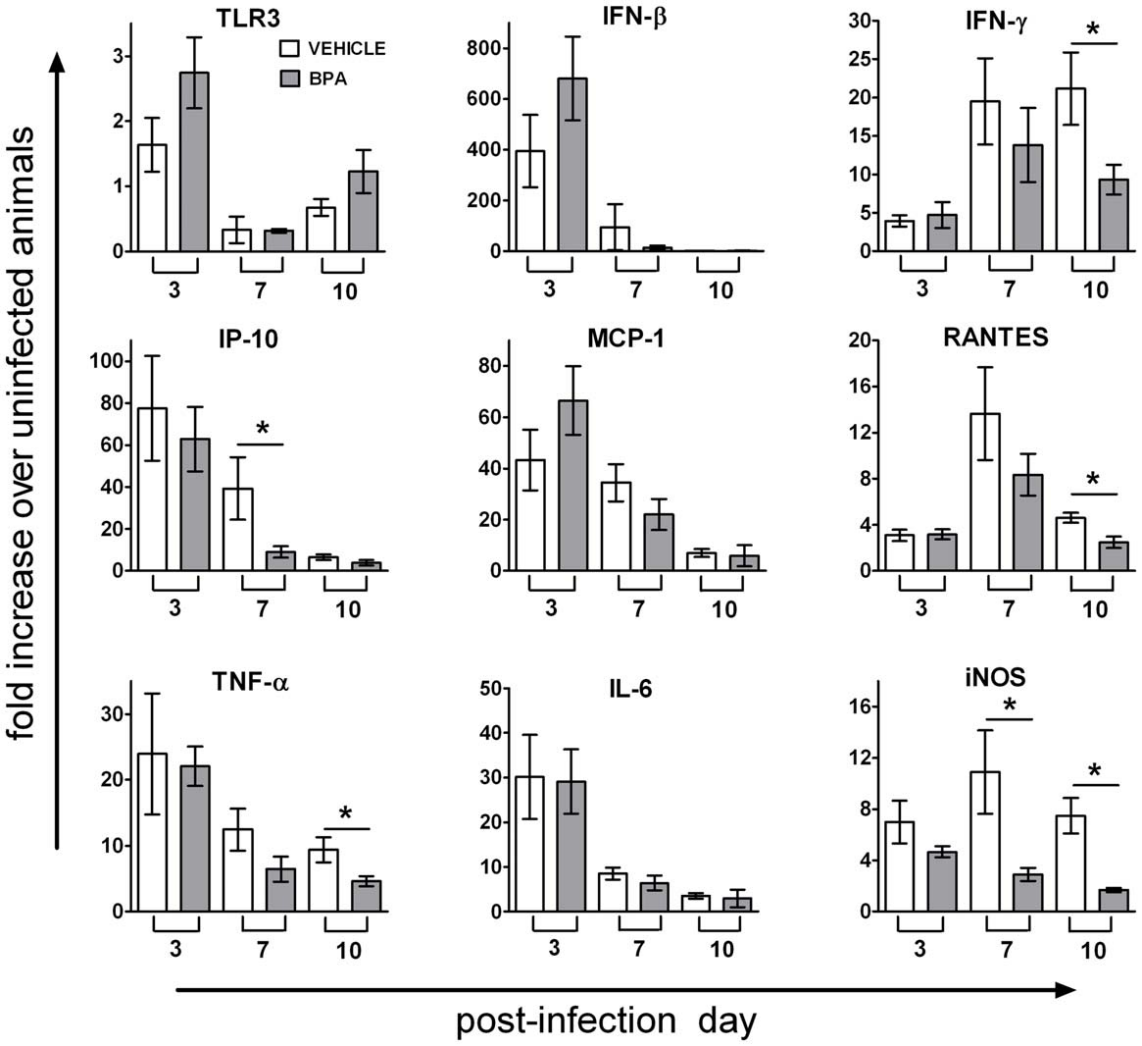
Gene expression in the lung following influenza virus infection. Adult offspring of the vehicle or BPA treated dams were infected with influenza A virus (HKx31) and lung tissues were harvested 0 (uninfected), 3, 7 or 10 days after infection. The levels of expression for the indicated genes were determined using qRT-PCR and normalized to the expression of gene for a ribosomal protein L13. The bar graphs depict the mean fold increase in the level of gene expression on different days following infection, relative to the level in uninfected animals from the same treatment group. BPA exposure did not affect the expression of any of these genes in naïve mice (not shown). Data represent mean ± SEM (n≥5/group); *p≤0.05.

The reduction in lung inflammation 7 days after infection suggests that there could be an attenuation of host antiviral immune responses in the lungs of mice that were maternally exposed to BPA. To further explore this possibility, the expression level of several key immunoregulatory genes was assessed 3, 7 and 10 days following infection. We observed a general pattern of reduced expression of genes for the cytokine TNF-α and the chemokines RANTES (CCL5), IP-10 (CXCL10), and MCP-1 (CCL2) in lungs of infected mice that had been exposed to BPA during development (Fig. 4), however, statistical significance of this reduction was only found for some of these genes. We also observed reduced expression levels of genes for interferon (IFN)-γ and inducible nitric oxide synthase (iNOS) in the lungs of BPA exposed mice (Fig. 4). This is intriguing because expression of iNOS and IFN-γ by innate immune cells, such as macrophages and neutrophils, influences lung pathology following influenza A virus infection [43]–[46]. On the other hand, this was not a global reduction in gene expression. Other genes, such as toll like receptor (TLR)-3, IFN-β and interleukin (IL)-6, were altered by influenza virus infection, but their level of expression was not modulated by developmental BPA exposure (Fig. 4).

### Developmental Exposure to BPA did Not Alter the Adaptive Immune Response following Influenza Virus Infection

**Figure 5:**
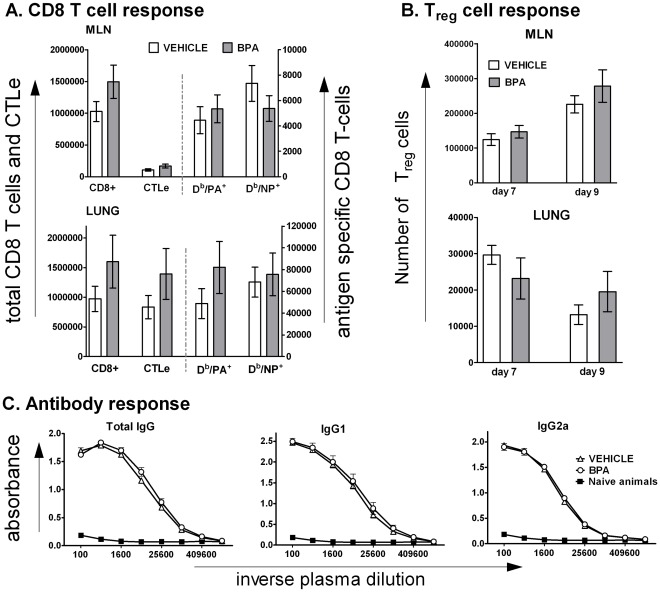
Mice maternally exposed to BPA mount an equivalent adaptive immune response to infection with influenza virus. Adult offspring of the treated dams were infected with influenza virus (HKx31). (A) Mice were sacrificed 9 days post-infection and immune cells were isolated from MLNs and lungs and stained with MHC class I-restricted tetramers and monoclonal antibodies, as described in the Material and Methods section. The number of CD3^+^CD8^+^ T cells (denoted as CD8^+^), CD3^+^CD8^+^CD44^hi^CD62L^lo^ cytotoxic effector CD8 T cells (denoted as CTLe), D^b^/PA_224–233_ and D^b^/NP_366–374_ specific CD8 T-cells (denoted as D^b^/PA^+^ and D^b^/NP^+^, respectively) were measured by flow cytometry. (B) Mice were sacrificed 7 or 9 days post-infection and immune cells were isolated from lungs and MLNs. The numbers of CD3^+^CD4^+^CD25^+^FoxP3^+^ T cells (T_reg_) were measured by flow cytometry. (C) A separate group of infected developmentally exposed mice were euthanized 14 days p.i., and blood was collected. The average amount of influenza virus-specific total IgG as well as virus-specific IgG_1_ and IgG_2a_ isotypes in plasma were measured using ELISA. Data shown are from adult female offspring; male offspring were also examined and findings were similar (data not shown). All data represent the mean (±SEM) of the total number of the specified cell types recovered from each organ (A-B) or from blood (C) of individual mice (n≥5/group/time point).

Despite reduced expression of several genes for key immunoregulatory molecules and a transient reduction in lung inflammation in the BPA-exposed group, influenza virus was almost completely cleared from the lungs by day 10 p.i. (Fig. 2). The critically important immune effectors responsible for the clearance of virus from the lung are virus-specific cytotoxic CD8^+^ T lymphocytes (CTL) [25], [26]. The CD8^+^ CTL response peaks about 9–10 days after HKx31 infection [24], [25]. To determine whether developmental exposure to BPA alters the CD8^+^ T cell response to influenza A virus infection, we isolated immune cells from lungs and the lung-draining MLN 9 days after infection. We did not detect any difference in the total number of CD8 T cells (CD3^+^CD8^+^), cytotoxic effector T cells (CTLe, CD3^+^CD8^+^CD44^hi^CD62L^low^) or virus antigen (NP or PA) specific CD8 T cells when we compared lung and MLN cells isolated from vehicle versus BPA exposed mice (Fig. 5A). Although the number increases due to infection, developmental exposure to BPA did not alter the number of CD4^+^ T cells, including those phenotypically defined as regulatory T cells (CD3^+^CD4^+^CD25^+^FoxP3^+^) in the MLN or lung on days 7 or 9 p.i. (Fig. 5B). These time points have been recently shown to reflect the peak and resolving response for regulatory T cells following primary influenza virus infection [47]. Total organ cellularity in MLN, lung, and spleen were also equivalent between two exposure groups (data not shown), as were the number of proliferating leukocytes, as detected by *in vivo* BrdU incorporation assays (data not shown). We also compared the level of influenza A virus specific antibodies in the circulation as another indicator of the adaptive immune response to infection. On day 14 p.i., the levels of virus-specific total IgG, as well as virus-specific IgG1 and IgG2a isotypes were similar between the two groups of mice (Fig. 5C).

### Maternal BPA Exposure did Not Influence Memory Immune Responses

**Figure 6:**
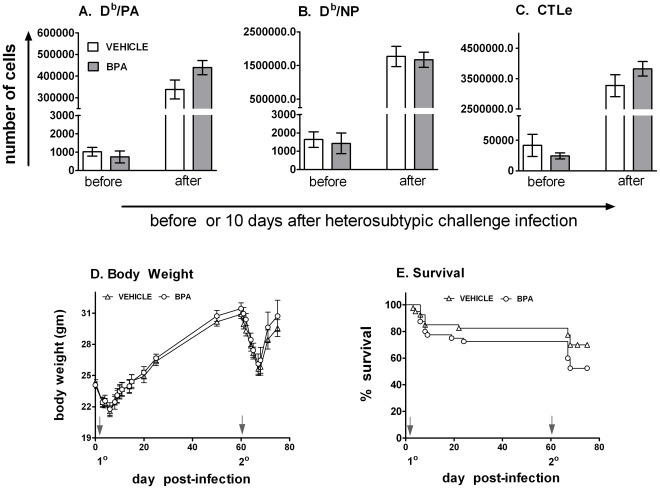
Developmental exposure to BPA does not adversely affect immune memory responses. Adult offspring of the treated dams were infected with influenza virus (HKx31, H3N2). Sixty days after infection with HKx31, mice were re-infected with a heterosubtypic strain of influenza A virus (PR8, H1N1). (A-C) Immune cells were isolated from lungs either before or 10 days after PR8 infection and stained with MHCI-restricted tetramers and monoclonal antibodies. The frequency of (A) influenza PA_224–233_ peptide specific (D^b^/PA^+^), (B) influenza NP_366–374_ peptide specific (D^b^/NP^+^) and (C) CD44^hi^CD62L^lo^ cytotoxic effector CD8 T cells (CTLe) were determined by flow-cytometry. The graphs represent the mean number (±SEM) of the indicated cell population recovered from infected lungs (n≥7/group). Similar results were obtained from cells isolated from spleen and MLNs (data not shown). (D,E) A separate group of developmentally exposed mice were monitored daily for (D) body weight loss and (E) survival from the day before primary (HKx31) infection, through to 20 days after secondary (PR8) infection. Data represent mean ± SEM (A-C, n>5/group/time point; D-E, n = 40/group). Statistical analyses were performed as described in the Material and Methods. There were no significant differences in the mean values between treatment groups.

Several reports suggest that early life exposure to certain pollutants reduces responses to routine childhood immunizations [48]–[50], suggesting that mechanisms controlling immunological memory are also potential targets of developmental toxicants. To test this, 60 days after primary infection with HKx31, mice were re-infected with a heterosubtypic strain of influenza A virus, PR8. We intentionally administered a dose of PR8 that was empirically determined to be lethal to naïve mice, but sub-lethal for immune competent mice with preexisting T cell based immunological memory. Using this strategy, we did not observe any difference in the number of differentiated effector CTL (CTLe) or virus-specific CD8^+^ T cells in the lung, MLN, or spleen 10 days after secondary infection (Fig. 6A-C). Moreover, the equivalent memory CD8 response correlated with equivalent disease outcomes, as demonstrated by similar body weight loss patterns and survival in both developmentally exposed groups (Fig. 6D,E). Consistent with these results, we did not observe any differences in the antibody response or lung inflammation in developmentally exposed mice that were given a second influenza virus infection (data not shown).

## Discussion

Infectious diseases remain among the major causes of illness and death worldwide. There is mounting evidence that developmental exposure to common chemicals from our daily environment represents an overlooked contributor to differences in host responsive capacity to infections [48]–[55]. Using the well characterized immune response that develops following influenza A virus infection, we created an assessment matrix that not only investigated the possible link between maternal exposure to BPA and altered host defenses against respiratory infections, but examined the effects of developmental exposure to BPA on several measures of innate and adaptive immune function known to play key roles in combatting infection. In many ways, our findings should be perceived as good news. Although concerning effects of low level exposure to BPA on reproductive cancers have been reported [56], [57], our findings indicate that the function of key cellular components of anti-viral adaptive immunity are largely unaffected by developmental exposure to BPA. Correlating with no change in the host’s ability to mount adaptive immunity against influenza virus infection, BPA exposure during development did not adversely affect host resistance to primary or recall infection. Based on these observation it appears that, in contrast to several other pollutants, such as dioxins and polychlorinated biphenyls, tobacco smoke, and lead [22], [23], [35], [49], [58]–[60], developmental exposure to BPA does not have significant influence on the magnitude or functional capacity of the antigen-specific adaptive component of the immune system.

Others have reported that early life BPA exposure correlated with an increase in lymphocyte functions, such as elevated Th2 cytokine production following a bacterial infection, or enhanced T cell and antibody responses following immunization with foreign proteins [17]–[19]. One possible explanation for these differing observations is that BPA acts on cellular mechanisms important for driving Th2-polarized, but not Th1-polarized, immune responses. That is, the effects of BPA on the immune system could mimic the effects of estrogens, by skewing the Th1/Th2 balance in favor of Th2 responses [61]–[63]. However, the idea that estrogens polarize toward Th2-biased responses is likely an oversimplification of the physiological and pharmacological effects of endogenous and exogenous estrogens on immune function [64], and therefore is probably not an adequate explanation. Other explanations for differences between our findings and prior reports include the dose administered, as well as the timing and route of exposure. Some of the studies in which altered immune function was reported used doses of BPA that were 10–100 fold higher. This may be a critical difference, as mice treated in our study had circulating BPA levels on par with levels reported in the general population. Moreover, in some of the other studies lower doses of BPA were used, and reported to have no effect on *ex vivo* immune cell functions or disease progression *in vivo* [17]–[19]. Similarly, direct exposure of mature animals to BPA, often in the mg/kg/day dose range, affected proliferation and cytokine production by *ex vivo* re-stimulated lymphocytes, but *in vivo* exposure in the µg/kg/day range did not alter these metrics or affect disease progression [65]–[67]. Given that there are reports of BPA exhibiting non-monotonic toxicity, is important to note that we also examined the consequences of maternal exposure to lower doses of BPA [0.5 and 5 µg of BPA/kg body weight/day] administered in the same dosing paradigm as the studies reported herein. We did not observe any differences in the primary immune response to influenza A virus infection in mice developmentally exposed to these doses of BPA (data not shown). Thus, the dose of BPA administered, particularly doses above 50 µg/kg/day, may modulate immune cell behavior, but there is no consensus on the relationship between doses to rodents in the mg/kg/day range and current human exposure.

Regulatory T cells can suppress the CD8 T cell response during virus infections [68]–[70]. It has been also reported that developmental exposure to BPA alters the magnitude of the regulatory T cell population [17], [18]. Using a transgenic mouse strain, one study showed that developmental exposure to BPA induced oral tolerance, and suggested that BPA might have effects on T cell selection and activation processes that involve clonal deletion and regulatory T cells [18]. However, in the current study, we did not observe an effect of developmental exposure to BPA on the number of CD4 T cells or CD4^+^CD25^+^FoxP3^+^ regulatory T cells in the MLN or lung. This finding is consistent with our observation that the overall CD8 T cell and antibody responses to infection did not differ between treatment groups. It is important to note that although the specific CD8 T cell population detected 9 days after infection may appear to be of greater magnitude in BPA exposed animals, no statistically significant differences in the mean percentage or number were observed in this or in independent repeats of this entire experiment. Thus, we conclude that the subtle changes observed in BPA exposed animals are unlikely to indicate a modification in the functional aspects of adaptive immunity that mediates virus clearance. In addition to the primary T cell response, our data also showed that developmental exposure to BPA did not compromise the ability to generate or execute an effective T cell based memory immune response. To our knowledge, this is the first demonstration of the consequences of developmental exposure to BPA on T cell based memory to an infection. Assessment of memory immune responses has implications beyond virus infections. For example, vaccines are intended to generate host protective immunological memory. An increased susceptibility to infection associated with exposure to environmental agents could result from reduced vaccine efficacy. Our data suggest that developmental exposure to BPA most likely would not interfere with efficacy of any vaccine intended for generating long-term T cell memory. Although we did not test antibody-based immune memory directly, our data show that development exposure to BPA had no effect on the ability of B cells to produce antibodies or undergo isotype class switching; from which we infer that a detrimental effect of early life exposure to BPA on antibody-based memory is also unlikely.

Although maternal BPA exposure impaired neither the magnitude nor the effectiveness of adaptive immunity, we did observe a reduction in pulmonary inflammation and cytokine expression levels in the lungs seven days following infection. This may also be associated with the variation seen in the lung virus titer of the mice. These observations suggest that developmental exposure to BPA may transiently diminish aspects of the inflammatory response to infection. Seven days post influenza virus infection in mice, two major lung infiltrating leukocyte populations are neutrophils and macrophages, and production of TNF-α and iNOS are hallmark functions of these cells [43]–[46]. Diminished expression of these genes in the infected lung suggests that developmental exposure to BPA might have some effects on inflammatory mediators, which can be produced by leukocytes or the structural cells of the lung. Several *in vitro* studies have shown that exposure to BPA diminishes functions of neutrophils and macrophages, including the production of TNF-α and nitric oxide [71]–[73]. An influence of maternal BPA exposure on cytokine production has also been reported in several animal models [17], [18]. Given that the level of inflammatory cytokines is associated with the physical malaise experienced during influenza virus infection [41], [42], it is possible that changes in cytokine production influence disease severity. However, using body weight as a measure of morbidity, we observed no evidence for this idea using two different strains of influenza virus. Interestingly, during the early days after infection, which is dominated by innate immune mediators, we observed slightly higher lung virus titers in some of the animals that were developmentally exposed to BPA. Although not statistically significant, this was observed in more than one study. Such a difference could be a consequence of reduced inflammation in the lung at that time, since early innate immune responses play a critical role in containing the virus during the early stages following infection [24]. At present we do not know whether the influence of BPA on lung inflammation is a result of direct effects of BPA on hematopoietic stem or progenitor cells that are exposed during development, or the result of an indirect effect of BPA, via endocrine or other systems that interact with hematopoietic or lung cells, and which could have been altered by developmental exposure to BPA. Moreover, whether and how early life exposure modulates diseases in which tissue inflammation plays a role in initiation of pathology remains to be determined. Our observations in the influenza-infected lung suggest that early life exposure may lead to attenuated rather than enhanced tissue inflammation.

To our knowledge, the current study is the first comprehensive investigation on the effects of developmental exposure to BPA on host resistance that integrates different aspects of immunity and quantitatively evaluates those parameters in a disease-specific manner. We demonstrate here that developmental exposure to low doses of BPA had only mild effects on the immune response to influenza A virus infection, and did not compromise the ability of the infected host to successfully clear the virus. Evidence exists that exposure to certain environmental agents can selectively affect some components of the immune system, and may even show pleiotropic effects on certain aspects of the immune response [22], [23], [52], [74]–[76]. Since the immune system is composed of multiple cell and tissue types, each having specific but overlapping contributions to overall host resistance, it is not surprising to observe subtle immunomodulation in one component of the antiviral response without an overall effect on virus clearance and disease outcome. Given that high doses of BPA have been shown to act as xenoestrogens *in-vitro* [6], [61], and considering the intricate relationship between the immune and endocrine systems, it is plausible that some BPA-mediated endocrine disruptive mechanism might be responsible for the transient depression in innate immunity that we observed following influenza virus infection. Alternatively, BPA could also have direct effects on cells that mediate innate immunity, such as down regulation of NF-κB pathways, as suggested by other *in vitro* studies [72]. Although the transient reduction in innate immunity did not negatively affect influenza virus clearance, in a different scenario wherein the inflammatory responses is critical for early pathogen clearance (e.g., certain bacterial infections), a reduction in innate immune functions from BPA exposure might prove detrimental. On the other hand, during innate immune-mediated diseases, such as chronic inflammation, BPA-mediated reductions in innate immune functions might have beneficial effects. While all these ideas are experimentally testable, the totality of our findings suggest that developmental exposure to low oral doses of BPA did not compromise the key aspects of anti-viral immunity to influenza virus infection.
